# Comparison of Sequential, Hybrid, and Quadruple Therapy Protocols in *Helicobacter pylori* Eradication: A Single-Center Study

**DOI:** 10.5152/eurasianjmed.2022.20360

**Published:** 2022-10-01

**Authors:** Ersin Kuloğlu, Bülent Albayrak, Hakan Dursun, Fatih Albayrak, Ömer Yılmaz

**Affiliations:** 1 Department of Internal Medicine, Atatürk University Faculty of Medicine, Erzurum, Turkey; 2 Department of Gastroentorology, Atatürk University Faculty of Medicine, Erzurum, Turkey

**Keywords:** Helicobacter pylori eradication, hybrid and quadruple therapy protocols

## Abstract

**Objective:**

Many treatment protocols are used in *Helicobacter pylori* eradication treatment within the framework of factors such as antibiotic resistance, drug side effects, patient compliance, and regional differences.

**Materials and Methods:**

: *H. pylori* was diagnosed with upper gastrointestinal system endoscopic biopsy in the Internal Diseases Gastroenterology Endoscopy Unit of Atatürk University Medical Faculty Hospital; a total of 229 patients over the age of 18 were evaluated prospectively by dividing them into 3 groups and applying 3 different *H. pylori* eradication treatment protocols.

**Results:**

A total of 229 patients who completed the treatment were included in the study. *H. pylori* eradication was achieved in 186 patients and not achieved in 43 patients. The *H. pylori* eradication success of our study was found to be 81.2%. Among the 84 patients in group 1, while *H. pylori* eradication was achieved in 67 of them, it was not achieved in 17 patients. The eradication success of quadruple treatment with bismuth was 79.8%. Also, among the 68 patients in group 2, while *H. pylori* eradication was achieved in 55 patients, it was not achieved in 13. The eradication success of the 14-day hybrid treatment was 80.9%. Among the 77 patients in group 3, while *H. pylori* eradication was achieved in 64 patients, it was not achieved in 13. The eradication success of the 10-day sequential treatment was 83.1%.

**Conclusion:**

It is necessary to conduct studies to find the most successful eradication regimen in primary care treatment of *H. pylori* in our country, to determine the regional antibiotic resistance rates, to individualize the proton pump inhibitor treatment due to metabolism and resistance differences, to examine the factors that stop from achieving the desired eradication success, and especially to avoid unnecessary antibiotic use.

**Main Points**

- *Helicobacter pylori* (HP) is a bacterium whose prevalence differs between developed and developing countries and is primarily associated with peptic ulcer disease, gastric adenocarcinoma, and gastric mucosal-associated lymphoma.
- The use of multiple antibiotics is required for the eradication of *H. pylori*, and besides the side effects of these antibiotics used, the increasing antibiotic resistance is still a current problem.
- Different eradication protocols may be recommended in the primary care treatment of HP in our region.
- It is obvious that culture studies are needed to prevent the unnecessary use of antibiotics

## Introduction

*Helicobacter pylori* (HP) is a bacterium with a varying prevalence among developed and developing countries which colonizes in the stomach roughly in half of the world’s population and is primarily associated with peptic ulcer disease, gastric adenocarcinoma, and gastric mucosal-related lymphoma.^1,2^ About the HP prevalence in our country, the overall prevalence of HP was 82.5% in the TURHEP study in 2003. In detecting HP, the gold standard may be defined as culture or histopathological examination.^3,4^ In the past decade, due to the resistance of HP to metronidazole and clarithromycin, the success rate reduced in triple treatment to less than 80%. Many treatment protocols are used in HP eradication treatment. Eradication rates between different protocols and in different regions of the same protocols vary in the framework of factors like antibiotic resistance, drug side effects, patient compliance, and regional differences.^5,6^

The purpose of the present study was to find the most appropriate treatment for HP by assessing the eradication rates of sequential treatment, hybrid treatment, and quadruple treatment protocols including bismuth.

## Materials and Methods

A total of 229 patients who were histopathologically diagnosed with HP with endoscopic biopsy of the upper gastrointestinal tract, who were over 18 years of age, who did not have gastric malignancy and gastric surgery, who had not previously received HP eradication treatment, who were not in pregnancy-lactation period, and who were without liver and renal insufficiency between 2017 and 2018 at Atatürk University, Medical Faculty Hospital, Department of Internal Medicine Gastroenterology Endoscopy Unit were divided into 3 groups and were then evaluated prospectively by giving 3 different HP eradication treatment protocols. Patients underwent upper gastrointestinal endoscopes by using Olympus CV-170, Olympus Evis Exera II CLV-180, and Olympus Evis Exera III CLV-190 video endoscopes under sedation anesthesia (propofol i.v.) and pharyngeal local anesthesia by experienced gastroenterologists. Multiple biopsies were taken from the antrum and corpus. The biopsy materials were placed in 10% formalin and sent to the pathology lab and were evaluated separately by experienced pathologists for HP inflammation activity, atrophy, and intestinal metaplasia according to the updated Sydney Biopsy Material Classification. The patients who had HP positivity as a result of the endoscopic biopsy were divided into 3 groups to evaluate the eradication rates of the 3 different treatments to be administered^7^ (Table 1). The study was approved by Atatürk University Faculty of Medicine Ethics Committee (ATA.09.06.17/31). An informed consent form was obtained from the patients included in the study for the examinations and treatment.

Quadruple treatment protocol with 14-day bismuth treatment was administered to the first group as bismuth subsalicillin tb 2 × 2 + pantoprazole 40 mg tb 2 × 1 + metronidazole 500 mg tb 3 × 1 + tetracycline 500 mg tb 4 × 1 (Table 2). The second group was given 14-day hybrid treatment protocol in the first 7 days: pantoprazole 40 mg tb 2 × 1 + amoxicillin 1 gr tb 2 × 1 and the following 7 days: pantoprazole 40 mg tb 2 × 1 + amoxicillin 1 gr tb 2 × 1 + clarithromycin 500 mg tb 2 × 1 + metronidazole 500 mg tb 3 × 1 (Table 2). The third group was given a 10-day sequential treatment protocol in the first 5 days: pantoprazole 40 mg tb 2 × 1 + amoxicillin 1 g tb 2 × 1 and the following 5 days: pantoprazole 40 mg tb 2 × 1 + clarithromycin 500 mg tb 2 × 1 + metronidazole 500 mg tb 2 × 1 (Table 2).

The pantoprazole treatment of the patients with gastric ulcers detected in endoscopy was completed in 8 weeks and the pantoprazole treatment of the patients with duodenal ulcer was completed in 6 weeks. 

All of the 229 patients who were included in the study completed the given treatments.

To evaluate the eradication after the treatment, patients underwent urea breath test or fecal antigen test after 6 weeks following the end of the current treatment at the latest. The Acro Rapid Test (Acro Biotech®, Inc., USA) antigen kits were used for fecal HP antigen test. 

For the urea breath test, after the patients took a deep breath, the basal breath sample was filled with a mouthpiece to the Helicobacter Test INFAI branded breathing bag on which the patient’s name was written. The patient was given 1 g citric acid with approximately 50-75 cc water. The kit that contained 75 mg C^13^ atom was then drunk by the patient with approximately 50-75 cc of water. After waiting for 30 minutes, the patient took a deep breath, and all of the patient’s breath was exhaled into the Helicobacter Test INFAI branded breathing bag. The difference between the values read at HeliFan Plus® (Fischer ANalysen Instrumente GmbH, Leipzig, Germany) device at basal (0th minute) and 30th minute and the result of the test were evaluated as positive if this value was over 4 per 1000 and negative if it was below 4 per 1000. 

### Statistical Analysis

The Statistical Package for the Social Sciences software 20.0 (IBM Corp., Armonk, NY, USA) program was used in the analysis of the data. Kruskal–Wallis test, 1-way variation analysis, and chi-square test were used to statistically compare the data. In the study, *P* < .05 was considered to be statistically significant.

## Results

Among the 229 patients who were included in the study, 114 were male (49.8 %) and 115 (50.2 %) were female. The mean age of the patients was 46.82 ± 13.33. The mean age of the patients was 44.26 ± 13.31 in group 1, 47.66 ± 13.36 in group 2, and 48.87 ± 13.03 in group 3. Among the patients in group 1, 40 were male and 44 were female, 36 were male and 32 were female in group 2, and 38 were male and 39 were female in group 3 (Table 1).

No statistically significant differences were detected between the groups in terms of age and gender (*P* = .074 and *P* = .809 for age and gender, respectively).

The rates of the side effects and the patients with side effects in all 3 treatment groups are given in Table 3. There was no need to end the treatment in any patient due to side effects.

All of the 229 patients who were included in the study completed the treatments. Eradication success rates were determined according to per-protocol analysis. *H. pylori* eradication was achieved in 186 patients and was not achieved in 43 patients. *H. pylori* eradication success rate of our study was found to be 81.2%. *H. pylori *eradication was achieved in a total of 67 of the 84 patients in group 1 but was not achieved in 17 patients. The eradication success of the bismuth quadruple treatment was 79.8%. *H. pylori* eradication was achieved in 55 of the 68 patients in group 2 but was not achieved in 13 patients. The eradication success of the 14-day hybrid treatment was 80.9%. *H. pylori *eradication was achieved in 64 of the 77 patients in group 3 but not in 13 patients. The eradication success of the 10-day sequential treatment was 83.1%. The eradication rates are shown in Table 4.

No statistically significant differences were detected according to the per-protocol analysis between the eradication rates of 14-day quadruple treatment, 14-day hybrid treatment, and 10-day sequential treatment (chi-square = 0.303; *P* = .86).

No statistically significant differences were detected between the gender and treatment responses of the 229 patients who were included in the study (*P* = .891). There were no statistically significant differences between the age and response to treatment in all patients (*P* = .916).

No statistically significant differences were detected between the endoscopic esophageal findings, gastric findings, and duodenal findings and response to treatment in all groups (*P* = .912, *P *= .608, and *P* = .272).

## Discussion

Although it varies regionally, multiple studies showed that resistance rates of antibiotics used to treat HP increased in all low, middle, and high-income countries in all regions of the world. In the Maastricht V Florence Declaration in 2015, the European HP Work Group recommended the bismuth quadruple treatment protocol (bismuth + proton pump inhibitor + tetracycline + metronidazole) as the first choice in areas where clarithromycin resistance is over 15%. The resistance rates of clarithromycin were approximately 30% in Italy and Japan, 50% in China, 15% in Sweden and Taiwan, and 10%-15% in the United States of America. The resistance rate of clarithromycin was approximately 40% in our country.^6,7^

The factors that cause failure in HP eradication are defined as regional antibiotic resistance, insufficient knowledge of the physician about the antibiotic resistance rates in the area where he/she applies the treatment, drug side effects, incompatibility of patients to treatment, not applying proper duration and dose of drugs, insufficient knowledge about cytochrome P450 2c19 polymorphism that regulates the proton pump inhibitor metabolism, increased bacterial load, smoking, and genetic diversity among HP strains. As there is a change of antibiotics in the treatment, the duration of successive treatment protocol can be confusing for patients and may affect treatment compliance.^5,6,8^

In the study conducted by Bakır et al^9^ to determine the sensitivity of 31 HP strains to various antibiotics in Marmara Region of our country, the resistance rates against clarithromycin, amoxicillin, metronidazole, tetracycline, and ciprofloxacin were found as 41.9%, 3.2%, 41.9%, 3.2%, and 45.2%, respectively. In areas where clarithromycin and metronidazole resistance are high (>15%), bismuth quadruple treatment is recommended in first-line treatment. Although metronidazole resistance has a negative effect on the effectiveness of bismuth quadruple treatment, it was not detected as deeply as clarithromycin resistance on classic triple treatment based on clarithromycin. Bismuth quadruple treatment administered for 10-14 days can achieve a treatment success of 85% and above even in areas with high metronidazole resistance. It is considered that the quadruple treatments that do not include bismuth (concomitant, hybrid, and sequential treatment) are more successful than classic triple treatment based on clarithromycin and are highly effective against clarithromycin resistance.^6,10^

In a study conducted by Harmandar et al^11^ in the Western Black Sea Region of our country consisted of a total of 160 patients in groups of 40 people who received 4 different treatments in the form of classic triple, 14-day bismuth quadruple treatment, 10-day sequential treatment, and 14-day sequential treatment, the eradication success rates were 70%, 82.5%, 92.5%, and 82.5%, respectively. Although the eradication success rate of 10-day sequential treatment was higher at 92.5% than quadruple treatment (82.5%), similar to our study, no statistically significant differences were detected between them in terms of treatment successes. 

The results of many studies and meta-analyses showed that the eradication rate of sequential treatment was >90% and was superior to standard triple treatment which had an eradication rate of <80%. When the results of a meta-analysis covering the data of 15 randomized controlled trials with more than 3000 patients were evaluated, it was seen that the success rate of the sequential treatment was 91.7% and that of the standard triple treatment was 76.7%.^12^

In a meta-analysis consisting of 11 studies on sequential treatment and standard treatment in HP infection, when the results of 2299 patients, 1143 of who received sequential treatment and 1156 of whom received 7-day standard treatment, were compared, it was found that the eradication success rate of the sequential treatment was reported to differ statistically significantly compared with 93.5% and 76.1%, respectively.^13^

In the meta-analysis study of Li et al^14^ in which 32 056 patients were analyzed for the treatment effect and 22 180 patients were taken for treatment tolerance analysis, HP eradication regimens were compared and the eradication rates of 14-day hybrid treatment, 10-14-day sequential treatment, and 10-14-day bismuth quadruple treatment were 89%, 87%, and 85%, respectively. No statistically significant differences were detected between the eradication success rates of these 3 treatment regimens.

Jung et al^15^ conducted a meta-analysis in Korea and included 21 eradication regimens that covered 43 studies comparing HP eradication treatments. The highest eradication success rate among all eradication regimens was detected in quinolone-based sequential treatment protocol with 91.4% in 14 days according to intention to treat (ITT) analysis. According to the ITT analysis, the eradication success rates of 7-day standard triple treatment, 10-day standard sequential treatment, 10- to 14-day hybrid treatment, and 10- to 14-day concomitant treatment were 71.1%, 76.2%, 79.4%, and 78.3%, respectively. In our study, the eradication rates of 14-day hybrid treatment and 10-day standard sequential treatment were viewed as higher compared to this study. 

In our study, the eradication success of bismuth quadruple treatment was found as 79.8%. Although this rate was close to 80%, which is the acceptable success rate, it was not the desired success rate. It is predicted that possible causes like high drug density in bismuth quadruple treatment, patients not taking their medications at the right time during the day, and patients skipping doses because of minor side effects of the drugs caused the eradication rate to be lower. There are many studies conducted in different countries with lower and higher eradication achievements than the 3 different treatment protocols that were given in our study. When the studies conducted in our country were evaluated, it was seen that regional antibiotic resistance rates changed. In this respect, regional differences were observed in the eradication rates of the bismuth quadruple treatment, sequential treatment, and hybrid treatment used in primary care treatment of HP in our country. 

In our study, the 90% desired eradication rate could not be achieved in all 3 different treatment protocols. In our study, among the reasons for this situation are unnecessary antibiotic use, antibiotic resistance, which is considered to have increased in the Eastern Anatolian Region, possible smoking of patients, and the presence of patients living in rural areas whose socioeconomic status was low.

The fact that our study consisted of 229 patients who were followed prospectively and that the eradication rates of bismuth quadruple treatment, sequential treatment, and hybrid treatment used in primary care treatment of HP were investigated and compared for the first time in Erzurum and surroundings are among the strong sides of our study. Among the 3 different eradication protocols given in our study, only the 14-day bismuth quadruple treatment remained below 80% with the acceptable eradication success rate of 79.8%. It was found that the 14-day hybrid and 10-day sequential treatment achieved an acceptable success rate. Although the eradication success rate of the bismuth quadruple treatment was close to the acceptable limit and due to the fact that it was not detected at an acceptable level and sequential and hybrid treatment did not reach the desired level of eradication rate of 90%, it may be recommended to use different eradication protocols in primary care treatment of HP in our region.

The shortcomings of our study are that the number of our cases is a little low, the smoking status is not known, and the inability to check for antibiotic resistance.

It is necessary to conduct studies to find the most successful eradication regimen in primary care treatment of HP in our country, to determine the regional antibiotic resistance rates, to individualize the proton pump inhibitor treatment due to metabolism and resistance differences, to examine the factors that stop from achieving the desired eradication success, and especially to avoid unnecessary antibiotic use.

## Figures and Tables

**Table 1.** Baseline Demographic and Clinical Characteristics of the Patients

**Characteristic**	**Group 1 (n = 84)**	**Group 2 (n = 68)**	**Group 3 (n = 77)**
**Gender**			
** Male**	40 (47.6%)	36 (52.9%)	38 (49.4%)
** Female**	44 (52.4%)	32 (47.1%)	39 (50.6%)
**Age (mean ± s.d.) years**	44.26 ± 13.31	47.66 ± 13.36	48.87 ± 13.03
*Helicobacter pylori* colonization*			
** Mild**	29 (34.6%)	22 (32.4%)	24 (31.2%)
** Moderate**	28 (33.3%)	23 (33.8%)	26 (33.7%)
** Marked**	27 (32.1%)	23 (33.8%)	27 (35.1%)

*Mild (+) small amount of colonization, moderate (++) a moderate amount of colonization, marked (+++) a large amount of colonization.

**Table 2.** The Number of Patient Groups and Treatment Protocols

	**Number (%)**	**Treatment Protocol**
**Group 1**	84 (36.7)	For 14 days: bismuth subsalicylate + pantoprazole + metronidazole + tetracycline
**Group 2**	68 (29.7)	For the first 7 days: pantoprazole + amoxicillin For the following 7 days: pantoprazole + amoxicillin + clarithromycin + metronidazole
**Group 3**	77 (33.6)	For the first 5 days: pantoprazole + amoxicillin; For the following 5 days: pantoprazole + clarithromycin 500 + metronidazole

**Table 3.** Side Effects

**Side Effects**	**Quadruplet Bismuth Treatment**	**Hybrid Treatment**	**Sequential Treatment**
Black color in feces	5	0	0
Taste impairment	3	0	0
Abdominal pain	5	3	2
Nausea	5	3	3
Diarrhea	3	2	1
Rate of patients with side effects	25%	11.7%	7.7%

**Table 4.** *Helicobacter pylori* Treatment Eradication Achievement Rates

	*Helicobacter pylori* Eradication Rates		Number
	(%)
**Group 1**	84	79.8
**Group 2**	68	80.9
**Group 3**	77	83.1

## References

[1] Eshraghian A Epidemiology of Helicobacter pylori infection among the healthy population in Iran and countries of the eastern Mediterranean Region: a systematic review of prevalence and risk factors. World J Gastroenterol. 2014;20(46):17618 17625. 10.3748/wjg.v20.i46.17618) 25516677 PMC4265624

[2] Hu Y Wan JH Li XY Zhu Y Graham DY Lu NH . Systematic review with meta-analysis: the global recurrence rate of Helicobacter pylori. Aliment Pharmacol Ther. 2017;46(9):773 779. 10.1111/apt.14319) 28892184

[3] Özaydin N Türkyılmaz SA Cali S . Prevalence and risk factors of helicobacter pylori in Turkey: a nationally-representative, cross-sectional, screening with the 13 C-urea breath test. BMC Public Health. 2013;13(1):1215. 10.1186/1471-2458-13-1215) PMC3880349 24359515

[4] Şimşek İ Binicier Ö . Helicobacter pylori. İç Hastalıkları Derg. 2011;18:13 26.

[5] Özden A HP’nin 30. Yılı (1983-2013) Helicobacter pylori Eradikasyonunda. Proton Pompa İnhibitörlerinin Yarattığı Mucize!! Güncel Gastroenteroloji. 2013;17(2):119 131.

[6] Malfertheiner P Megraud F O'morain CA et al. Management of Helicobacter pylori infection—the Maastricht V/Florence consensus report. Gut. 2017;66(1):6 30. 10.1136/gutjnl-2016-312288) 27707777

[7] Özden A Proton Pompa İnhibitörlerinin Helicobacter pylori Enfeksiyonu Tedavisindeki Etkinlikleri. Güncel Gastroenteroloji. 2014;18(1):59 66.

[8] Gale SD Erickson LD Brown BL Hedges DW . Interaction between Helicobacter pylori and latent toxoplasmosis and demographic variables on cognitive function in young to middle-aged adults. PLoS ONE. 2015;10(1):e0116874. 10.1371/journal.pone.0116874) PMC4295891 25590622

[9] Bakir Ozbey SO Ozakin C Keskin M . Antibiotic resistance rates of Helicobacter pylori isolates and the comparison of E-test and fluorescent in situ hybridization methods for the detection of clarithromycin resistant strains. Mikrobiyol Bul. 2009;43(2):227 234. 19621607

[10] Chey WD Leontiadis GI Howden CW Moss SF . ACG clinical guideline: treatment of Helicobacter pylori infection. Am J Gastroenterol. 2017;112(2):212 239. 10.1038/ajg.2016.563) 28071659

[11] Harmandar F Helicobacter Pylori Eradikasyonunda Sequential treatmentnin Etkinliği [Yandal Uzmanlık Tezi]. Zonguldak: Karaelmas Üniversitesi Tıp Fakültesi; 2012. (Danışman. Prof. Dr. Yücel Üstündağ).

[12] Park HG Jung MK Jung JT et al. Randomised clinical trial: comparative study of 10-day sequential treatment with 7-day standard triple treatment for Helicobacter pylori infection in naïve patients. Aliment Pharmacol Ther. 2012;35(1):56 65. 10.1111/j.1365-2036.2011.04902.x) 22066530

[13] Tong J Ran Z Shen J et al. Sequential treatment vs. standard triple therapies for Helicobacter pylori infection: a meta-analysis. J Popul Ther Clin Pharmacol. 2009;34(1):41 53. 10.1111/j.1365-2710.2008.00969.x 19125902

[14] Li BZ Threapleton DE Wang JY et al. Comparative effectiveness and tolerance of treatments for Helicobacter pylori: systematic review and network meta-analysis. BMJ. 2015;351:h4052. 10.1136/bmj.h4052) PMC4541168 26290044

[15] Jung YS Park CH Park JH Nam E Lee HL . ­Efficacy of Helicobacter pylori eradication therapies in Korea: a systematic review and network meta-analysis. Helicobacter. 2017;22(4):e12389. 10.1111/hel.12389) 28425141

